# Generalized reconfigurable memristive dynamical system (MDS) for neuromorphic applications

**DOI:** 10.3389/fnins.2015.00409

**Published:** 2015-11-03

**Authors:** Mohammad Bavandpour, Hamid Soleimani, Bernabé Linares-Barranco, Derek Abbott, Leon O. Chua

**Affiliations:** ^1^Department of Electrical and Computer Engineering, University of California, Santa BarbaraSanta Barbara, CA, USA; ^2^Department of Bioengineering of Imperial CollegeLondon, UK; ^3^Instituto de Microelectrónica de Sevilla, IMSE-CNM, Universidad de Sevilla and CSICSevilla, Spain; ^4^School of Electrical and Electronic Engineering, The University of AdelaideAdelaide, SA, Australia; ^5^Department of Electrical and Computer Engineering, University of California, BerkeleyBerkeley, CA, USA

**Keywords:** general cellular mapping, hybrid memristor-crossbar/CMOS platform, FitzHugh-Nagumo (FHN) neuron model, Adaptive Exponential (AdEx) integrate and fire neuron model, Izhikevich neuron model, dynamical behavior analysis

## Abstract

This study firstly presents (i) a novel general cellular mapping scheme for two dimensional neuromorphic dynamical systems such as bio-inspired neuron models, and (ii) an efficient mixed analog-digital circuit, which can be conveniently implemented on a hybrid memristor-crossbar/CMOS platform, for hardware implementation of the scheme. This approach employs 4*n* memristors and no switch for implementing an *n*-cell system in comparison with 2*n*^2^ memristors and 2*n* switches of a Cellular Memristive Dynamical System (CMDS). Moreover, this approach allows for dynamical variables with both analog and one-hot digital values opening a wide range of choices for interconnections and networking schemes. Dynamical response analyses show that this circuit exhibits various responses based on the underlying bifurcation scenarios which determine the main characteristics of the neuromorphic dynamical systems. Due to high programmability of the circuit, it can be applied to a variety of learning systems, real-time applications, and analytically indescribable dynamical systems. We simulate the FitzHugh-Nagumo (FHN), Adaptive Exponential (AdEx) integrate and fire, and Izhikevich neuron models on our platform, and investigate the dynamical behaviors of these circuits as case studies. Moreover, error analysis shows that our approach is suitably accurate. We also develop a simple hardware prototype for experimental demonstration of our approach.

## 1. Introduction

The human nervous system is an intriguing complex system capable of performing intricate tasks using an enormous number of neurons each connected via synapses to several thousand neighboring neurons. Mathematical modeling of biological neural elements such as neurons, synapses, and glial cells has been a long standing active research area (Hodgkin and Huxley, 1952; Fitzhugh, 1961; Abbott, 1999; Izhikevich, 2003; Brette and Gerstner, 2005). Recently, the hardware implementation of neural networks has been the subject of much research with three motivations: (1) exploitation of functional mechanisms of biological neural systems; (2) development of neural prosthetics (i.e., replacement of a biological neural system by an electronic circuit), and building an artificial brain as a long-term goal; and (3) development of artificial neural networks for use in engineering applications. Two main factors in this research area are accuracy (targeting biological plausibility) and computational complexity of the model and system (targeting efficiency in terms of the large scale simulation and implementation cost). In general, there is a trade-off between model accuracy and its computational complexity. Different platforms have been applied for hardware realization of neuromorphic dynamical systems. Here, we investigate main approaches for this challenge:

1. Special purpose computing architectures have been developed to simulate neurobiological networks and functions using their specially designed software tools for large scale simulations (Ahmadi and Soleimani, 2011; Furber et al., 2014; Minkovich et al., 2014). Even though these systems are flexible and biologically realistic with considerably high performance due to their massively parallel architecture, the presented hardware approaches are based on bulky and power-hungry workstations with relatively high cost and development time. Hence, these approaches are often impractical for public access, and general purpose large-scale simulations.
2. Analog CMOS platform is considered to be a significant choice for direct implementation of neural dynamic functions (Linares-Barranco et al., 1991; Arthur and Boahen, 2011; Wijekoon and Dudek, 2012; Soleimani et al., 2014). However, this approach is not a general approach. This implies that the structure of the circuit must be modified if there are any changes to the starting equations. Moreover, non-linear functions in target mathematical models are directly implemented using inherent non-linearity of the circuit elements and networks. Hence, the variability and mismatch of the circuit elements drastically affect the circuit performance, and implementation of some models with special non-linear dynamics is cumbersome and intractable. Besides, this hardware platform is comparatively inflexible, and model adjustment is typically troublesome in these circuits.
3. A digital platform is used to realize bio-inspired neural cells. Most digital approaches (Weinstein et al., 2007; Soleimani et al., 2012, 2015; Cassidy et al., 2013) use digital computational units to implement the mathematical equations describing the behavior of biological neural cells. Another type of digital approach (Matsubara and Torikai, 2013) is presented based on Cellular Automata (CA) consisting logic gates, register arrays, and reconfigurable wires, and the dynamics of the target system are determined by the wire patterns. This platform can be further subcategorized into FPGA based (Weinstein et al., 2007; Soleimani et al., 2012, 2015; Matsubara and Torikai, 2013) and digital custom IC based (Cassidy et al., 2013) hardware, where FPGAs provide more configurability and lower development time, but at a higher cost in terms of area, power, and speed in comparison with the digital custom ICs. Generally, a digital platform achieves rapid development time, high reconfigurability, and immunity to device mismatch. However, its silicon area and power consumption is directly related to the mathematical complexity of the model, and it is not beneficial for complex models due to excessive utilization of high-cost computational units such as comparators, multipliers, and adders. Moreover a CA based approach (Matsubara and Torikai, 2013) uses a complicated wiring network that occupies a large switching area, decreases the maximum frequency of the overall system clock by making a long critical path, and results in a cumbersome implementation process.
4. Full custom analog/digital (mixed mode) implementations that comprise low-power, fast analog circuits and programmable, mismatch immune digital circuits. Generally, for this approach, neural computation is performed in the analog domain while the communication of spikes between nervous cells is carried out in the digital domain (Schemmel et al., 2008; Benjamin et al., 2014; Moradi and Indiveri, 2014). Recently using this approach, we have proposed a Cellular Memristive Dynamical System (CMDS; Bavandpour et al., 2014) consisting a dynamical systems cellular mapping, and a memristor-based hardware platform for implementing a wide range of neuromorphic systems. The mixed analog-digital nature of this system results in accuracy and efficiency in terms of hardware implementation cost. However, in this platform, the number of memristors employed in a two-dimensional system has a quadratic relation with the number of cells where an *n*-cell system requires 2*n*^2^ memristors. So, the area consumption is significantly increased with the number of cells. Besides, the circuit provides just one-hot digital values of the dynamical variables, which constrains the networking schemes, so an additional circuit is needed to provide the analog value of the variables.

Our approach is a cellular-based system that discretizes dynamical variables resulting in a cellular phase plane, stores the equilibrium curves in a memristive crossbar-based analog memory block, evaluates the velocity and direction of the vector field in the cells, and tracks the state point in the space using VCOs and pointer registers. This system is a fully reconfigurable general approach capable of implementing a wide range of two dimensional neuromorphic dynamical systems such as FitzHugh-Nagumo (FHN; Fitzhugh, 1961), Adaptive Exponential (AdEx) integrate and fire (Brette and Gerstner, 2005), and Izhikevich neuron models (Izhikevich, 2003).

One promising technology particularly suited for analog and mixed analog-digital computing is based on hybrid circuits that integrate CMOS and memristor devices (Strukov and Likharev, 2006; Bahar et al., 2007; Kavehei et al., 2012). The memristor is a two-terminal thin-film device whose resistance can be tuned in a nonvolatile and analog way (Chua, 1971; Strukov et al., 2008; Yang et al., 2008). In the context of analog and mixed circuit applications, recent advances in memristive devices and their integration with CMOS enable efficient implementations of nanoscale analog-grade resistive elements that can be fine-tuned after fabrication (Alibart et al., 2012). The proposed approach is an efficient generalized system with high reconfigurability due to using memristor-crossbar/CMOS hardware platform. This circuit is highly programmable because reconfiguration is carried out by adjusting memristance values, while the structure of the system remains fixed. It implies that a wide range of target dynamical systems such as various neuron models can be easily programmed into the system and their parameters can be easily adjusted without changing the hardware structure. Furthermore, the circuit can accurately mimic different dynamic neuronal behaviors, and yield a variety of biological-like spike trains as well as single spike shapes.

The implementation constraint has strongly limited the power of dynamical systems in modeling, and neuroscientists have been unable to develop an accurate model. Moreover, a number of behaviors such as special output signal shapes have no elegant analytical description. Our approach significantly alleviates the limitation of computational effort in dynamical functions.

The rest of the paper is organized as follows: In Section 2, we discuss the proposed general mapping scheme. The memristor-crossbar/CMOS based hardware structure of the proposed platform is introduced in Section 3. Section 4 presents implementation of three widely used neuron models called the FHN neuron model (Fitzhugh, 1961), AdEx neuron model (Brette and Gerstner, 2005), and Izhikevich neuron model (Izhikevich, 2003) based on the proposed approach, and investigates various dynamical responses of the resultant circuits as the case studies. Section 5 introduces design steps and practical results of a simple hardware prototype of our approach. The error analysis of the proposed approach is studied in Section 6, and the networking and learning capability of the system is explained as two remarks in Section 7. Finally, the work is concluded in Section 8.

## 2. General cellular mapping for neuromorphic dynamical systems

The first step toward developing a memristor-crossbar/CMOS hardware for implementing a reconfigurable neuromorphic dynamical system is drawing a proper mapping to represent target dynamical systems. This step plays a significant role in the whole design process because the proposed mapping ought to satisfy a number of crucial conditions: It ought to have the capability of (1) transforming the relatively complicated equations of neuromorphic dynamical systems into a number of simplified fully implementable equations, (2) obtaining a maximum accuracy considering the available hardware resources for implementation, (3) being applied to a wide range of neuromorphic dynamical systems, and (4) bringing reconfigurability into the hardware by separating the variable features of the neuromorphic dynamical systems (requisite information in phase plane for reconstructing vector field) from the shared features (computations for reproducing time-domain signals), and implementing the variable features on the reconfigurable memristive part of the circuit.

In this section, we propose a cellular mapping that properly satisfies the aforementioned conditions. In Section 2.1, basic concepts of dynamical systems in neuroscience are reviewed, and a general form for two dimensional neuromorphic dynamical systems and its simplified forms are presented. One of the simplified forms, which covers most of the two dimensional neuron models, is the basis of our mapping in the next subsections. In Section 2.2, a two dimensional cellular space is defined and the mapping equations for transforming the target dynamical system from the continuous space to the cellular space is presented. Then, according to the simplified form, a technique for calculating the motion velocity of the state point in each cardinal direction in the cellular space is proposed. Applying this technique to the simplified general form in the cellular space results in a mapping which satisfies first, third and fourth out of four above-mentioned conditions. But the second condition related to the timing and asynchrony ought to be satisfied in order to maximize the accuracy of tracking state point in the cellular space. In Section 2.3, the concept of timing and asynchrony in our cellular approach is explained, the requisite timing condition is introduced, and the final satisfactory timing equations and cell change policy based on the timing parameters are presented.

### 2.1. Dynamical systems in neuroscience

In computational modeling of neural cell behaviors and phenomena, neuroscientists aim to point out critical features and factors as the system variables and parameters, and draw out deterministic laws governing the evolution of these variables over time (Gerstner and Kistler, 2002). These laws are presented by mathematical functions, and the collection of resultant equations is called a dynamical system. Dynamical systems model the behavior of a given system without a thorough comprehensible knowledge on all the governing rules for its evolution. This feature significantly matches the nature of neuroscience, and turns dynamical system into a strong mathematical tool for effective modeling of individual components in the nervous system.

In neuromorphic modeling, two-dimensional dynamical systems are popular because of their capability of phase portrait representation. The overall qualitative dynamics of a system can be easily investigated through the study of the phase portrait of the system including different types of equilibrium points and curves and consequently local velocity vectors in the phase space, and also a geometric representation of special trajectories determining topological behaviors of trajectories in a neighborhood in the phase space. Hence, several neuromorphic models are presented in the general form described as:

(1) $$\{\begin{array}{l} \frac{dx}{dt}=F(x,y)+b\\ \frac{dy}{dt}=G(x,y)+c \end{array}$$

where *F* and *G* are smooth functions, *b* and *c* are input variables and a number of auxiliary functions on *x*,*y* may be attached. In this case, the phase plane is a two-dimensional space, (*x, y*) ∈ *Z*^2^ represents the location of state point in the plane, and (ẋ, ẏ) (velocity vector) determines the velocity and direction of the motion. According to Equation (1), the terms *b* and *c* directly influence the velocity of the moving state point in both dimensions. Here, we temporarily ignore *b* and *c* (*b* = *c* = 0) in the general form to simplify developing the mapping scheme, and then apply their influences directly on the velocity vector in the last step of the hardware design.

In most neuromorphic dynamical systems with the general form of Equation (1) the equilibrium lines are single-valued functions. This implies that one dynamical variable is a single-valued function of another one. Hence, the simplified general form of neuromorphic dynamical systems, ignoring input parameters *b* and *c*, can be rewritten as one of these four different forms:

(2) $$\{\begin{array}{l} \frac{dx}{dt}=\alpha \cdot (F(x)-y)\\ \frac{dy}{dt}=\beta \cdot (G(x)-y) \end{array}$$

(3) $$\{\begin{array}{l} \frac{dx}{dt}=\alpha \cdot (F(y)-x)\\ \frac{dy}{dt}=\beta \cdot (G(x)-y) \end{array}$$

(4) $$\{\begin{array}{l} \frac{dx}{dt}=\alpha \cdot (F(x)-y)\\ \frac{dy}{dt}=\beta \cdot (G(y)-x) \end{array}$$

(5) $$\{\begin{array}{l} \frac{dx}{dt}=\alpha \cdot (F(y)-x)\\ \frac{dy}{dt}=\beta \cdot (G(y)-x) \end{array}.$$

Note that our approach supports all four different above-mentioned forms, but considering this fact that most of the popular neuron models such as the FitzHugh-Nagumo (Fitzhugh, 1961), Izhikevich (Izhikevich, 2003), and AdEx (Brette and Gerstner, 2005) neural models, fit into the first simplified general form represented in Equation (2), we continue the rest of the paper based on this general form.

### 2.2. Cellular space and generalized mapping

**Figure 1:**
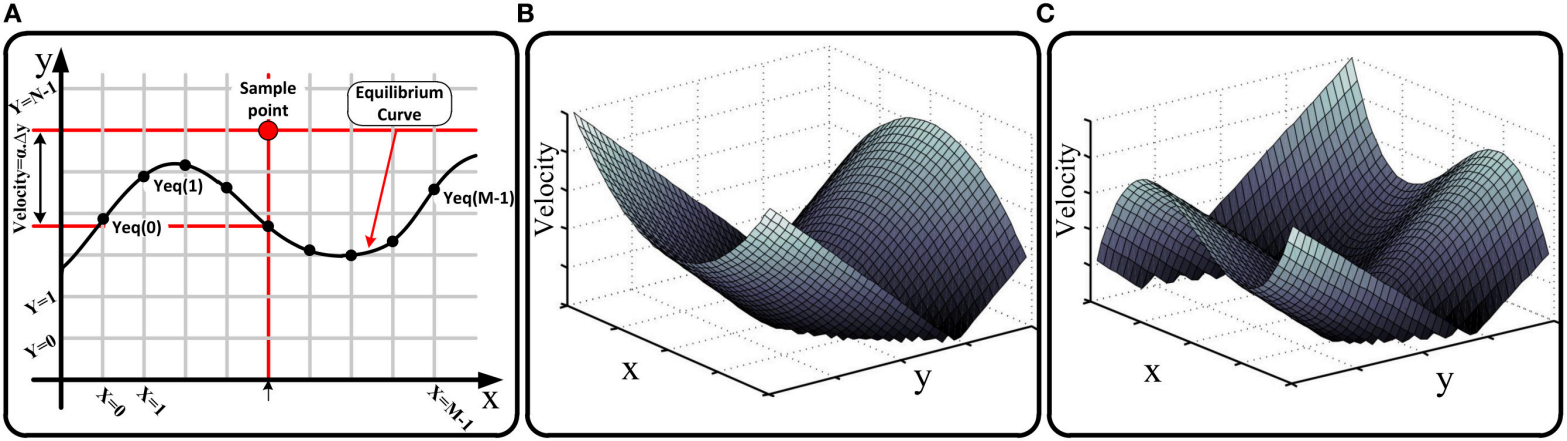
**(A)** Cellular phase portrait of a sample dynamical system, one of its equilibrium curves, and evaluating the velocity of a sample point using equilibrium curve according to Equation (9), **(B)** absolute value of the velocity in the phase plane for a quadratic equilibrium curve used in Izhikevich neuron model (Izhikevich, 2003), **(C)** absolute value of the velocity in the phase plane for a cubic parabola equilibrium curve used in FHN (Fitzhugh, 1961) neuron model.

In the first step, we map the continuous phase plane to a cellular plane. As shown in Figure 1A, in the cellular space, discrete points on the state variable axes and accordingly discrete mesh-like points on the phase plane are considered. Thus, *X* = *i* where *i* ∈ ***M*** ≡ {0, 1, …, *M* − 1}, and *Y* = *j* where *j* ∈ ***N*** ≡ {0, 1, …, *N* − 1} are discrete variables parallel to *x* and *y* in the continuous space. The location of state point in the ***M*** × ***N*** cellular space can be evaluated as:

(6) $$\left\{ \begin{array}{l} X=\left\lfloor \frac{x-x_{\text{min}}}{\Delta x}\right\rfloor \\ Y=\left\lfloor \frac{y-y_{\text{min}}}{\Delta y}\right\rfloor \end{array}\right.$$

where $\Delta x=\frac{x_{\text{max}}-x_{\text{min}}}{M}$ and $\Delta y=\frac{y_{\text{max}}-y_{\text{min}}}{N}$ are cellular steps in each dimension. In the next step, which is one of the key steps in the mapping scheme, we evaluate the velocity and direction of the vector field in the cells in the way that it can be easily implemented on our proposed hardware platform. In CMDS approach (Bavandpour et al., 2014), the value of velocity and direction in each cell is directly stored in its corresponding memory cell in a cellular memory block, which requires embedding a memory block of size 2 · *N* · *M* memory cells (memristors) in the hardware implementation. Here, we significantly shrink the size of memory block using a technique to evaluate the motion velocity and direction in the simplified general form.

As mentioned, the first simplified general form represents the most common condition in neuromorphic dynamical systems where *y* is a function of *x* in both equations. In this condition, equilibrium curves of the system (considering $\frac{dx}{dt}=0,\frac{dy}{dt}=0$) are *y*_eqx_ = *F*(*x*) and *y*_eqy_ = *G*(*x*). In the cellular representation, we have:

(7) $$\{\begin{array}{l} Y_{\text{eqx}}(X)=F(x_{\text{min}}+X\cdot \Delta x)\\ Y_{\text{eqy}}(X)=G(x_{\text{min}}+X\cdot \Delta x) \end{array}$$

where *Y*_eqx_ and *Y*_eqy_ are two arrays of *M* real numbers presenting the value of the *y* variable for discrete points on the equilibrium curves corresponding to different discrete values of *X*. We refer to these arrays as the equilibrium arrays in the cellular space. Figure 1A shows the cellular phase plane and a sample equilibrium curve in the plane. As shown, the value of *y* variable for cross points of equilibrium curve with *X* = *i* (*i* = 0, 1, …, *M* − 1) vertical lines are the elements of *Y*_eq_ array. It will be substantiated that we can evaluate the velocity vector (Ẋ, Ẏ) for all cells in the phase plane just using the equilibrium arrays. Consider a sample point (*X* = *i, Y* = *j*); the velocity vector for this point is calculated as:

(8) $$\{\begin{array}{l} \dot{X}(X,Y)=\alpha \cdot (F(x_{\text{min}}+X\cdot \Delta x)-(y_{\text{min}}+Y\cdot \Delta y))\\ \dot{Y}(X,Y)=\beta \cdot (G(x_{\text{min}}+X\cdot \Delta x)-(y_{\text{min}}+Y\cdot \Delta y)) \end{array}.$$

Substituting Equation (7) in Equation (8), the velocity vector equation is given by:

(9) $$\{\begin{array}{l} \dot{X}(X,Y)=\alpha \cdot (Y_{\text{eqx}}(X)-(y_{\text{min}}+Y\cdot \Delta y))\\ \dot{Y}(X,Y)=\beta \cdot (Y_{\text{eqy}}(X)-(y_{\text{min}}+Y\cdot \Delta y)) \end{array}.$$

As the above equation shows, the velocity vector depends on the equilibrium arrays and a number of predetermined parameters. In other words, the absolute value of the velocity at every state point is proportional to its absolute vertical distance (distance in *y* direction) from the equilibrium curve, and its direction (forward/backward) is proportional to the position of the state point (above/below) to the equilibrium curve in the phase plane. The equations of cell change policy based on the timing parameters are represented in the next subsection after defining these parameters and drawing requisite timing and asynchrony conditions in our approach. Figures 1B,C are shown the absolute value of the velocity in the phase plane for two applied equilibrium curves in the neuron models. For hardware realization of our approach, we store the equilibrium arrays on the memristor crossbar and evaluate the velocity vector using hardware structure of Equation (9). Clearly, the resultant circuit is fully reconfigurable because the equilibrium arrays can be evaluated and programmed on the memristive memory block once a new dynamical system is presented.

### 2.3. Timing and asynchrony in cell change policy

According to the previous subsection, the velocity of motion and the motion direction (forward/backward in each dimension) are variable in the cellular phase plane, and can be evaluated using Equation (9). Note that the evaluated velocities are in units of (1∕s) where *x* and *y* are unitless quantities. For uniform representation in the cellular space, we ought to evaluate the cellular velocity with the unit of (cell∕s). So, the velocity values are divided by the amount of cellular steps:

(10) $$\{\begin{array}{l} {\dot{X}}_{\text{cellular}}(X,Y)=\frac{\dot{X}(X,Y)}{\Delta x}\\ {\dot{Y}}_{\text{cellular}}(X,Y)=\frac{\dot{Y}(X,Y)}{\Delta y} \end{array}.$$

Here, with the aim to simplify the timing analysis, we convert the concept of motion velocity to the concept of motion time. Hence, the cellular motion time (the amount of time required for moving one cell) can be obtained by reversing the cellular motion velocity as:

(11) $$\{\begin{array}{l} T_{x}^{\left(X,Y\right)}=\text{NF}(\frac{1}{{\dot{X}}_{\text{cellular}}})\\ T_{y}^{\left(X,Y\right)}=\text{NF}(\frac{1}{{\dot{Y}}_{\text{cellular}}}) \end{array}$$

where NF(·) is a function presenting restrictions imposed by hardware implementation that limits the time delay value in a specific boundary. A simple form for this boundary function can be represented as:

(12) $$\text{NF}(\text{input})=\{\begin{array}{ll} \text{MAX} & \text{input}>\text{MAX}\\ \text{input} & \text{MIN}\leq \text{input}\leq \text{MAX}\\ \text{MIN} & \text{input}<\text{MIN} \end{array}$$

where MIN and MAX are lower and upper boundaries. Clearly, our cellular approach is asynchronous, so the state variables are changed asynchronously. Considering this fact, it is crucial to proportionally handle the cellular motion time in one direction when other state variable is changed and consequently causes a change in motion time in both directions. In other words, time handling of the motion in each dimension is independent, but the motion in one dimension influences the motion time in another dimension by changing the vertical distance of the state point from the corresponding equilibrium curve, and it ought to be considered.

Here, we illustrate the time handling procedure for a special case, and then derive a generalized rule to handle the two dimensional timing. Assume that the initial state (*t* = 0) of the system is (*i, j*) and the corresponding motion times are $T_{x}^{\left(i,j\right)}$ and $T_{y}^{\left(i,j\right)}$ where $\left|T_{x}^{\left(i,j\right)}|<|T_{y}^{\left(i,j\right)}\right|$. At $t=|T_{x}^{\left(i,j\right)}|$ the state point moves to (*i* + 1, *j*) and the motion time of variable *X* is updated to $\left|T_{x}^{\left(i+1,j\right)}\right|$, but this is not reasonable to update the motion time of variable *Y* to $\left|T_{y}^{\left(i+1,j\right)}\right|$ without taking into account the elapsed time in point (*i, j*) resulting in no change in *Y* direction. Hence, the passed time is considered as a portion of the new motion time, and deducted from this time amount:

(13) $$T_{y}^{\left(i+1,j\right)}(\text{new})=(1-\frac{\left|T_{x}^{\left(i,j\right)}\right|}{\left|T_{y}^{\left(i,j\right)}\right|})\cdot T_{y}^{\left(i+1,j\right)}.$$

Regarding above explanations, an overall recursive rule can be derived to handle the dimensional motion times for moving from any given cell *P* ∈ ***M*** × ***N*** to its neighbor cell *Q* ∈ ***M*** × ***N***:

(14) $$t_{r}^{x}(Q)=\{\begin{array}{ll} \left(1-\frac{t_{r}^{y}(P)}{t_{r}^{x}(P)})\cdot |T_{x}^{\left(Q\right)}\right| & X(Q)=X(P)\\ \left|T_{x}^{\left(Q\right)}\right| & X(Q)\neq X(P) \end{array}$$

(15) $$t_{r}^{y}(Q)=\{\begin{array}{ll} \left(1-\frac{t_{r}^{x}(P)}{t_{r}^{y}(P)})\cdot |T_{y}^{\left(Q\right)}\right| & Y(Q)=Y(P)\\ \left|T_{y}^{\left(Q\right)}\right| & Y(Q)\neq Y(P) \end{array}$$

where $t_{r}^{x}\left(Q\right)$ and $t_{r}^{y}\left(Q\right)$ are, respectively, the remaining time to change for *X* and *Y* state variables at *Q* cellular point which are decreased with passing operation time of the system. The cell change policy is given by:

(16) $$X(t^{+})=\{\begin{array}{ll} X(t)+\text{sign}(T_{x}^{\left(X(t),Y(t)\right)}) & t_{r}^{x}=0\\ X(t) & \text{Otherwise} \end{array}$$

(17) $$Y(t^{+})=\{\begin{array}{ll} Y(t)+\text{sign}(T_{y}^{\left(X(t),Y(t)\right)}) & t_{r}^{y}=0\\ Y(t) & \text{Otherwise} \end{array}.$$

Thus, when the shorter remaining motion time for the faster state variable is elapsed, the state variable is changed by one cell, and the remaining motion time for the other state variable is updated according to the motion time vector in the new state and using Equation (13). In the following sections, it is qualitatively and quantitatively shown that the dynamical behaviors of a given two dimensional dynamical system described generally by Equation (2) can be accurately mimicked by evaluating velocity vector given by Equation (9) using the equilibrium arrays, and following the rules of motion given by Equations (14–17).

## 3. Memristive circuit implementation

The memristor is a nano-scale passive variable resistor with memory whose resistance changes depending on the polarity and magnitude of a voltage applied to the device terminals and the duration of this voltage application. Threshold condition is one of the key characteristics of the memristor. Based on this feature, small voltages across the memristor, below its threshold (*V*_th_), do not cause a considerable memristance change, while larger voltages, greater than the threshold, induce much greater memristance changes (Jo et al., 2010). In this approach, we use the memristors as pre-programmed analog memories, so their memristance values ought to remain constant during operation time of the circuit. This implies that the maximum applied voltage to the memristors ought to be lower than the threshold voltage.

The emerging technology of memristive circuits is one of the most promising technologies for hardware implementation of computational systems. Accordingly, developing new analog computation approaches compatible with a hybrid memristor-crossbar/CMOS platform is a significant ongoing research area (Bichler et al., 2012; Ligang et al., 2013; Merrikh-Bayat et al., 2013). In this paper, we engage this platform in the area of neuromorphic engineering and bio-modeling by developing a novel mapping scheme for neuromorphic dynamical systems, which makes them compatible for implementation on the aforementioned platform. In this section, we present the hardware structure of the system according to the proposed mapping scheme in the previous section.

**Figure 2:**
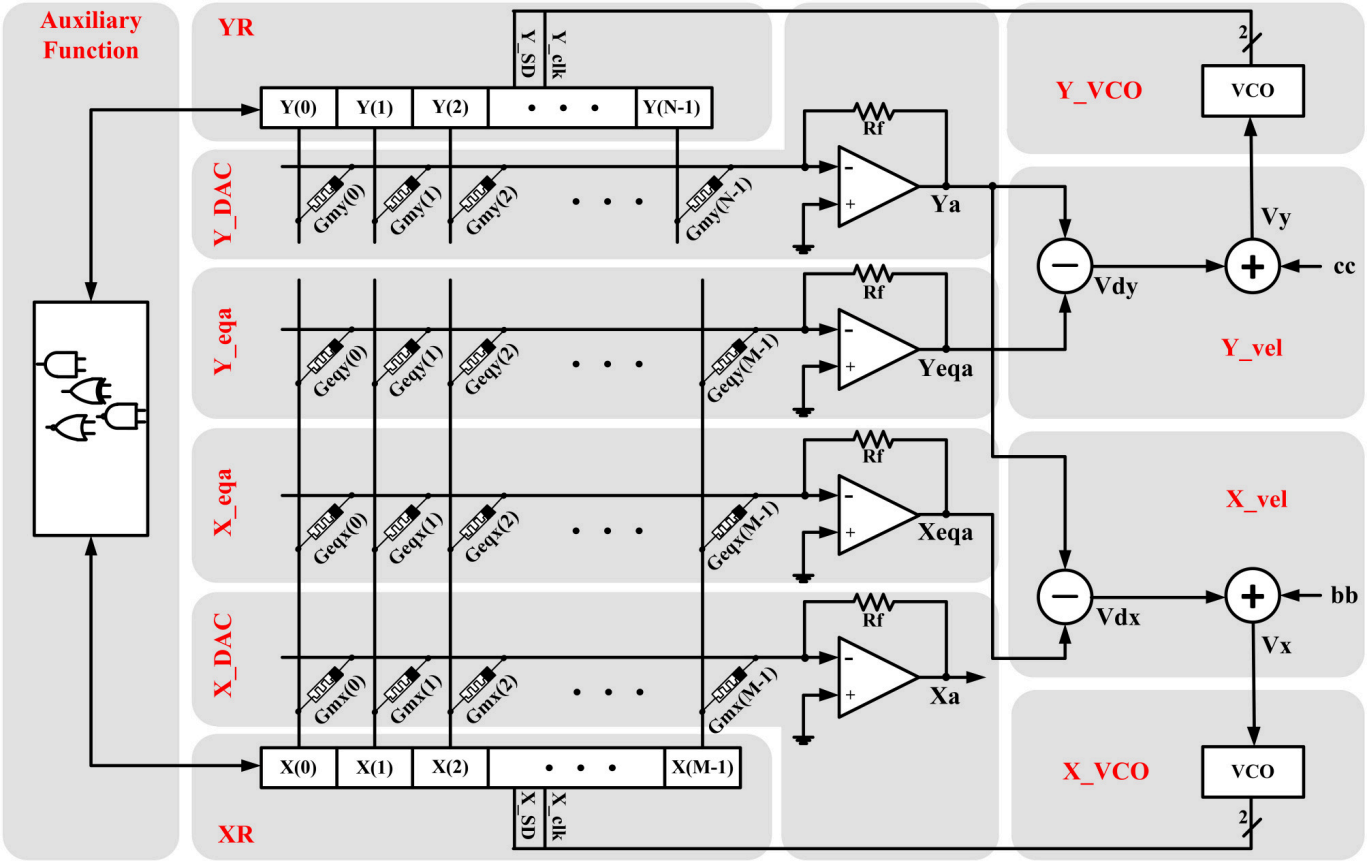
**Hardware structure of the proposed memristive dynamical circuit which is totally compatible with the hybrid memristor crossbar/CMOS platform because the memristor crossbar part of the circuit is isolated from the CMOS-based part**.

The proposed memristive circuit is shown in Figure 2. In this circuit, the memristors are connected in a crossbar architecture which offers flexibility, scalability, and simplicity, and also provides maximum density. Moreover, the memristor crossbar is separated from the CMOS-based part of the circuit causing the compatibility of the circuit for implementation on the promising hybrid memristor-crossbar/CMOS technology. As shown in the figure, the circuit is broken down to different parts which are explained one by one as follows:

### 3.1. XR and YR

In the proposed circuit, *X* and *Y* discrete variables are, respectively, stored in two *M*-bit and *N*-bit bidirectional shift registers named XR and YR using one-hot encoding described as:

(18) $$\text{XR}(i)=\{\begin{array}{ll} 1 & X(t)=i\\ 0 & \text{Otherwise} \end{array},i=0,1,\ldots ,M-1$$

(19) $$\text{YR}(i)=\{\begin{array}{ll} 1 & Y(t)=i\\ 0 & \text{Otherwise} \end{array},i=0,1,\ldots ,N-1.$$

Clearly in this representation, increasing or decreasing one unit in the *X* and *Y* discrete variables are, respectively, equivalent to one-bit forward or backward shift in the registers. Also, registers can be reset through a control signal.

### 3.2. X_DAC and Y_DAC

These units receive the one-hot value of the discrete variables from XR and YR and produce their proportional analog values in the outputs Xa and Ya. The circuit diagram of these units contains an opamp-based multi-input analog adder where the input resistors are replaced by the memristors, so the gain of each input can be controlled by changing the value of its input memristance. Thus, as the Figure 2 shows, we set the memristances proportional to the weight array of the input bits in the one-hot encoding, which is an array of linearly increasing real numbers. Thus, the governing equation of these circuits can be given by:

(20) $$\{\begin{array}{l} \text{Xa}=-\Sigma_{i=0}^{M-1}\text{XR}(i)\cdot G_{\text{mx}}(i)\cdot R_{f}\cdot v_{d}\\ \text{Ya}=-\Sigma_{i=0}^{N-1}\text{YR}(i)\cdot G_{\text{my}}(i)\cdot R_{f}\cdot v_{d} \end{array}$$

where *R*_*f*_ is the feedback resistor of the op-amps, *v*_*d*_ is the voltage level from digital registers representing logic “one." In this circuit, reading conductance of the memristors ought not to affect their value. On the other hand, it is mentioned that if the applied voltage to the memristor is below its threshold (*V*_th_), it does not induce considerable change in the memristance. Hence, as an essential condition, the applied voltage to the memristors ought to be lower than the threshold value (*v*_*d*_ < *V*_th_). The conductance values functions *G*_mx_(·) and *G*_my_(·) for the memristors are described as:

(21) $$\{\begin{array}{l} G_{\text{mx}}(i)=(A_{x}.i+1)\cdot G_{\text{mx0}}\\ G_{\text{my}}(i)=(A_{y}.i+1)\cdot G_{\text{my0}} \end{array}$$

where *A*_*x*_, *A*_*y*_ are the normalizing coefficients and *G*_mx0_, *G*_my0_ are the initial conductance parameters. For uniformity, the output voltage range for both units ought to be clamped in $\left(v_{\text{min}}^{\text{op}},v_{\text{max}}^{\text{op}}\right)$. For satisfying lower boundary condition of this interval, we have:

(22) $$\begin{array}{l} v_{\text{min}}^{\text{op}}=G_{\text{mx0}}\cdot R_{f}\cdot v_{d}=G_{\text{my0}}\cdot R_{f}\cdot v_{d}\\ \;\Rightarrow G_{\text{mx0}}=G_{\text{my0}} \end{array}$$

and for satisfying upper boundary condition, we have:

(23) $$\begin{array}{l} \;v_{\text{max}}^{\text{op}}=(A_{x}\cdot (M-1)+1)\cdot G_{\text{mx0}}\cdot R_{f}\cdot v_{d}\\ \;\;\;\;\;\;\;\;\;=(A_{y}\cdot (N-1)+1)\cdot G_{\text{mx0}}\cdot R_{f}\cdot v_{d}\\ \;\Rightarrow \frac{A_{x}}{A_{y}}=\frac{N-1}{M-1}. \end{array}$$

The parameters can get various values satisfying the conditions described in Equations (22) and (23). Note that there is an undesirable negative sign multiplied in the output voltages Xa and Ya in Equation (20), but it is not the source of any difficulties in our approach, because what is calculated in the next blocks is just the difference of the voltages and the negative sign can be easily handled.

### 3.3. X_eqa and Y_eqa

In these units, the equilibrium arrays given by Equation (7) are stored on the memristors, and the analog output voltages proportional to the equilibrium values for current YR state is produced in the *X*_eqa_ and *Y*_eqa_ outputs. Thus, the governing equation of these circuits can be given by:

(24) $$\{\begin{array}{l} X_{\text{eqa}}=-\Sigma_{i=0}^{M-1}\text{XR}(i)\cdot G_{\text{eqx}}(i)\cdot R_{f}\cdot v_{d}\\ Y_{\text{eqa}}=-\Sigma_{i=0}^{N-1}\text{XR}(i)\cdot G_{\text{eqy}}(i)\cdot R_{f}\cdot v_{d} \end{array}$$

where *R*_*f*_ is the feedback resistor of the op-amps, *v*_*d*_ is the voltage level from digital registers representing logic “one,” and the conductance values functions *G*_eqx_(·) and *G*_eqy_(·) for the memristors are described as:

(25) $$\{\begin{array}{l} G_{\text{eqx}}(i)=(A_{y}\cdot \frac{Y_{\text{eqx}}(i)-y_{\text{min}}}{\Delta y}+1)\cdot G_{\text{my0}}\\ G_{\text{eqy}}(i)=(A_{y}\cdot \frac{Y_{\text{eqy}}(i)-y_{\text{min}}}{\Delta y}+1)\cdot G_{\text{my0}} \end{array}.$$

Similar to the equations of the X_DAC, Y_DAC units presented in Equation (20), there is an undesirable negative sign in the expression for the output voltages *X*_eqa_ and *Y*_eqa_ in Equation (24), but it is not the source of any difficulties. This is because voltage differences are calculated in the next blocks and the sign can be easily handled.

### 3.4. X_vel and Y_vel

As shown in Figure 2, each of these units contains one analog voltage subtractor and one analog voltage adder which can be merged in one op-amp based circuit. The subtractor receives the proportional analog value of equilibrium arrays (*X*_eqa_ or *Y*_eqa_) and the *Y* state variable (Ya), and produces the proportional analog value of the velocity (*V*_dx_ or *V*_dy_) using Equation (9). The governing equations of the subtractor circuits are:

(26) $$\{\begin{array}{l} V_{\text{dx}}=G_{\text{sx}}\cdot (X_{\text{eqa}}-\text{Ya})\\ V_{\text{dy}}=G_{\text{sy}}\cdot (Y_{\text{eqa}}-\text{Ya}) \end{array}$$

where *G*_sx_ and *G*_sy_ are the gain of subtractors. Note that the coefficients α and β in Equation (9), and the aforementioned undesirable negative sign from the previous steps ought to be considered in the gain of the subtractors in this step. In the next step, the adders add the proportional analog voltages of the *b* and *c* parameters in Equation (1), which were ignored (*b* = *c* = 0) for the sake of convenience in the mathematical process and hardware design, to the output of subtractors to apply their influence directly to the velocity of moving state point. The adder equations are given by:

(27) $$\{\begin{array}{l} V_{x}=V_{\text{dx}}+\text{bb}\\ V_{y}=V_{\text{dy}}+\text{cc} \end{array}.$$

For adjusting the mathematical parameters *b* and *c* with their proportional analog voltage value bb and cc, they are then multiplied by the adjusting coefficients as:

(28) $$\{\begin{array}{l} \text{bb}=G_{b}\cdot b\\ \text{cc}=G_{c}\cdot c \end{array}$$

where *G*_*b*_ and *G*_*c*_ are the adjusting parameters. We will determine the essential conditions on how all the introduced parameters in X_vel and V_vel units can be chosen, after introducing the VCO units and their parameters.

### 3.5. X_VCO and Y_VCO

These units contain VCOs and controller circuit. The VCO receives the analog voltage corresponding to the velocity (*V*_*x*_ or *V*_*y*_) and produces the linearly proportional motion clock pulse, which is used for clocking the shift of the XR and YR registers, with a specific range of the frequency. Functional equation of the VCOs is given by:

(29) $$f_{\text{out}}^{\text{Xclk}}(V_{x})=\{\begin{array}{ll} f_{\text{max}} & |V_{x}|>\text{UT}\\ G_{\text{vco}}\cdot |V_{x}| & \text{LT}<|V_{x}|\leq \text{UT}\\ f_{\text{min}} & |V_{x}|\leq \text{LT} \end{array}$$

(30) $$f_{\text{out}}^{\text{Yclk}}(V_{y})=\{\begin{array}{ll} f_{\text{max}} & |V_{y}|>\text{UT}\\ G_{\text{vco}}\cdot |V_{y}| & \text{LT}<|V_{y}|\leq \text{UT}\\ f_{\text{min}} & |V_{y}|\leq \text{LT} \end{array}$$

where *G*_vco_ is the VCO coefficient, the UT and LT are, respectively, the upper-hand and lower-hand input voltage threshold of the VCOs and *f*_max_ and *f*_min_ are, respectively, the maximum and minimum output frequencies of the VCOs. The VCOs produce two asynchronous clock pulse signals based on the derived equation on their output pins X_clk and Y_clk. Note that the VCOs inherently satisfy Equations (14) and (15).

The controller circuit determines the direction of the motion (shift direction of the XR and YR state registers) on the pins X_SD and Y_SD. Zero logical value on these pins means that the next change in their proportional state registers is a backward shift, and the one logical value means that the next change is a forward shift:

(31) $$\text{X}\_\text{SD}=\{\begin{array}{ll} 1 & V_{x}>0\\ 0 & \text{otherwise} \end{array}$$

(32) $$\text{Y}\_\text{SD}=\{\begin{array}{ll} 1 & V_{y}>0\\ 0 & \text{otherwise} \end{array}.$$

Eventually, the state registers are shifted at the positive edge of their proportional clock pulse signals X_clk and Y_clk:

(33) $$\text{XR}(t_{\text{posedge}}^{+})\{\begin{array}{ll} \text{XR}\gg 1 & \text{XR}(M-1)\neq 1,\text{X}\_\text{SD}=1\\ \text{XR}\ll 1 & \text{XR}(0)\neq 1,\text{X}\_\text{SD}=0\\ \text{XR} & \text{XR}(M-1)=1,\text{X}\_\text{SD}=1\\ \text{XR} & \text{XR}(0)=1,\text{X}\_\text{SD}=0 \end{array}$$

(34) $$\text{YR}(t_{\text{posedge}}^{+})\{\begin{array}{ll} \text{YR}\gg 1 & \text{YR}(N-1)\neq 1,\text{Y}\_\text{SD}=1\\ \text{YR}\ll 1 & \text{YR}(0)\neq 1,\text{Y}\_\text{SD}=0\\ \text{YR} & \text{YR}(N-1)=1,\text{Y}\_\text{SD}=1\\ \text{YR} & \text{YR}(0)=1,\text{Y}\_\text{SD}=0 \end{array}$$

where $t_{\text{posedge}}^{+}$ is the time when a positive edge is appeared on the input clock pulse of the state registers.

As we mentioned before, we ought to draw the essential conditions on the coefficients for proper operation of the system. According to the Equation (2), the analog velocity of the state point in *x* direction is α times of its absolute analog vertical distance (distance in *y* direction) from the equilibrium curve, and in *y* direction is β times of its absolute analog vertical distance from the equilibrium curve. Accordingly, in the cellular space, the cellular velocity of the state point in *x* direction is $\alpha \cdot \frac{\Delta y}{\Delta x}$ times of its absolute cellular vertical distance from the equilibrium curve, and in *y* direction is β times of its absolute cellular vertical distance from the equilibrium curve. Note that in the cellular space, the cellular velocity is equal to the frequency of VCOs. So, for a special condition where the vertical distance of the state point from the equilibrium curve is equal to one cell, the cellular output frequencies of the VCOs are $f_{\text{out}}^{\text{Xclk}}=\alpha \cdot \frac{\Delta y}{\Delta x}$ and $f_{\text{out}}^{\text{Yclk}}=\beta$. In this condition, if we merge the Equations (21), (24–26), (29), and (30) (assuming *b* = *c* = 0) to evaluate the output frequency of the VCOs, we have:

(35) $$\{\begin{array}{l} A_{y}\cdot G_{\text{my0}}\cdot R_{f}\cdot v_{d}\cdot G_{\text{sx}}\cdot G_{\text{vco}}=\alpha \cdot \frac{\Delta y}{\Delta x}\\ A_{y}\cdot G_{\text{my0}}\cdot R_{f}\cdot v_{d}\cdot G_{\text{sy}}\cdot G_{\text{vco}}=\beta \end{array}.$$

By dividing these two equations, we obtain:

(36) $$\frac{G_{\text{sx}}}{G_{\text{sy}}}=\frac{\alpha \cdot \Delta y}{\beta \cdot \Delta x}.$$

Now, assume the special condition where the vertical distance of the state point from the equilibrium curve is equal to zero and *b* = Δ*x, c* = Δ*y*. In this condition, the frequencies of the VCOs are $f_{\text{out}}^{\text{Xclk}}=f_{\text{out}}^{\text{Yclk}}=1$. According to the Equations (28–30), we have:

(37) $$\{\begin{array}{l} G_{b}\cdot \Delta x\cdot G_{\text{sx}}=1\\ G_{c}\cdot \Delta y\cdot G_{\text{sy}}=1 \end{array}.$$

By dividing these two equations, we have:

(38) $$\frac{G_{b}}{G_{c}}=\frac{\beta }{\alpha }.$$

Therefore, the parameters must satisfy the essential conditions given by Equations (35–38).

Auxiliary Function: This unit is a logical block for implementing the auxiliary functions like threshold conditions and resetting functions.

The proposed circuit is significantly more efficient in term of area in comparison with the previous similar approach named CMDS (Bavandpour et al., 2014) while achieving the same performance and accuracy. In the Table 1, our approach is compared with the CMDS in terms of circuit elements/blocks for implementing a two-dimensional 100 × 100 cellular dynamical system.

**Table 1:** **Hardware assessment of the proposed approach in comparison with the CMDS approach for implementing a two-dimensional 100 × 100 cellular dynamical system**.

**Circuit element/block**	**Proposed approach**	**CMDS**
Memristor	400	20,000
Switch	–	400
Analog adder	1	1
VCO	2	2
Auxiliary function	1	1
State variable registers	2	2
Analog output	Yes	No
One-hot digital output	Yes	Yes

## 4. Dynamical behavior analysis

The behavior of a biological neuron depends on many intrinsic and extrinsic factors, such as the morphology of its dendritic tree, the type and characteristics of ion-gated channels and voltage expressed by the neuron, the location of the stimulating input, and so on. These factors are indeed important, because they determine not only the neuronal response but also the rules that govern dynamics of the neuron. Accordingly, different neuron models are developed to mimic different responses of the neuron and their related dynamics (bifurcations). Our generalized circuit can mimic the dynamics and the various responses of a wide range of neuron models with different computational complexity. In this section, we investigate three main neuron models as the FHN (Fitzhugh, 1961), AdEx (Brette and Gerstner, 2005), and Izhikevich neuron models (Izhikevich, 2003), and show that the circuit is able to properly mimic different responses of different neuron models.

### 4.1. FitzHugh-Nagumo (FHN) neuron model

The FHN is a two-dimensional neuron model derived form the simplified Hodgkin-Huxley (HH) model for biological process of spike generation in squid large axons (Fitzhugh, 1961). This model is considered as a relatively complex mathematical model due to a third power factor in its equations. FHN is represented by a simplified set of equations as:

(39) $$\{\begin{array}{l} \dot{v}=v-\frac{v^{3}}{3}-u+I\\ \dot{u}=a\cdot (v+0.7-0.8u) \end{array}$$

where *v* and *u* are, respectively, the membrane potential variable and the recovery variable, *I* is the injected stimulus current, and *a* is an adjustable parameter. As shown, FHN model utilizes no auxiliary resetting function to reproduce spiking behaviors. Hence, it does not employ the auxiliary function block in the circuit implementation. Mathematical analysis of this model simply shows that this system behaves like a relaxation oscillator. It implies that if the stimulus input current of the model exceeds a specific threshold, the model will follow a characteristic trajectory in phase space before the state variables relax back to their resting values. Typically, this behavior is how a biological neuron generates a spike (defined as a short and fast elevation of membrane potential) after injecting a short stimulus input current. Comparing the FHN model with the general form of target systems presented in the previous section, it can be rewritten in the general form of Equation (2) and then mapped easily on the proposed platform. The general form of FHN neuron model in our mapping is given by:

(40) $$\{\begin{array}{l} x=v,y=u\\ \alpha =1,\beta =0.8a\\ b=I,c=0\\ F(x)=x-\frac{v^{3}}{3}\\ G(x)=(x+0.7)/0.8 \end{array}.$$

Our FHN-MDS approach can exhibit all significant qualitative phenomena of the original FHN model and their underlying bifurcations. In this study, four main bio-inspired phenomena of FHN model are individually investigated on our approach.

#### 4.1.1. Absence of all-or-none spike

**Figure 3:**
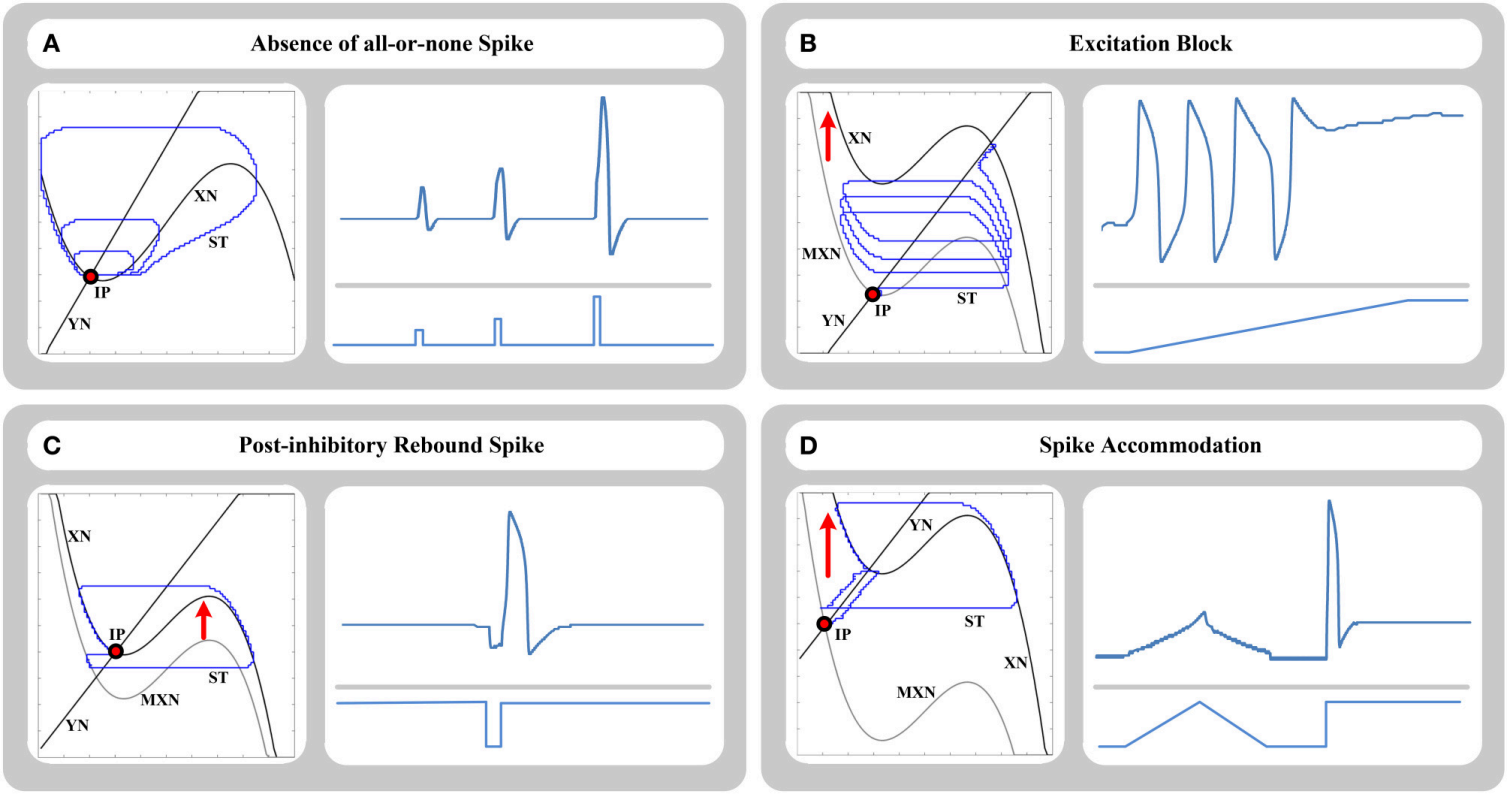
**Phase-plane (left side) and time-domain (right side) representations of (A) absence of all-or-none spikes phenomenon, (B) excitation block phenomenon, (C) post-inhibitory rebound spike phenomenon, and (D) spike accommodation phenomenon, for 64-bit FHN-MDS neuron circuit**. In the phase plane, XN and YN are the nullclines, MXN is the momentary location of XN nullcline, ST is the trajectory of the state point in the plane, and IP is the initial location of the state point.

The original FHN model is capable of producing the absence of all-or-none spikes as it is produced in HH model in response to stimulus current *I*. According to this behavior, the amplitude of output relaxation trajectory is directly and continuously related to the amplitude of the injected current pulse *I* to the model. Weak stimulation produces a small-size relaxation trajectory as a subthreshold response. Stronger stimulation produces an intermediate-size trajectory as a partial-size spike, and strong stimulation produces a large-size trajectory as a supra-threshold firing response. Figure 3A shows time-domain and phase-plane representations of this phenomenon produced by our approach. As shown, size of trajectories in the phase plane is directly related to the amplitude of applied input pulses in the time domain.

#### 4.1.2. Excitation block

According to this response, the neuron stops repetitive firing and goes back to a stable resting state as the amplitude of the input current increases. This type of response is based on a special bifurcation scenario in the phase plane representation. When input current *I* is relatively weak, the equilibrium point (the point that the nullclines intersect) is located on the left branch of X-nullcline resulting in a stable state, and hence the model is in the resting state. As the input current increases, the nullcline shifts upward in the phase plane, so the equilibrium point moves onto the middle branch of the nullcline resulting in an unstable state. Hence, the model produces a limit cycle in the phase plane and starts a repetitive firing in the output. Further increase of the stimulus current shifts the equilibrium point to the right branch of the N-shaped nullcline resulting in a stable state again, so the previous firing state is blocked. The FHN-MDS approach can exhibit this behavior as accurate as the original FHN model. Transition from the resting state to the repetitive firing, and then the blocking state produced by the FHN-MDS approach using a ramp input current are shown in Figure 3B in both phase and time domains.

#### 4.1.3. Post-inhibitory rebound spike

Another basic behavior of the original FHN model is post-inhibitory rebound spikes. This phenomenon is also called anodal break excitation. This behavior is produced in response to application of a short negative pulse to the model. As the negative pulse is applied, hyperpolarization is occurred, and the resting state slides to the left. At the moment when negative pulse is finished, anodal break is occurred, stable equilibrium point promptly shifts up, and the state point makes a transitory large-amplitude excursion to move from previous location of the stable point to its current location and rest. This transient state results in a single spike in time domain. This response is investigated on the FHN-MDS circuit in Figure 3C, and the results show that the approach can accurately produce this behavior.

#### 4.1.4. Spike accommodation

This type of response is a common basic dynamical mechanism produced by HH-family models. According to this response, slow increase of the injected current *I* to a specific amplitude does not cause a firing behavior, while prompt increase of the current to the same (even smaller) amplitude results in a spike in neuron output. In the case of gradual stimulation, the stable resting point of the model gradually slides to the right side of the phase plane, and the state point smoothly follows it without any additional excursion such as a firing cycle. In the case of prompt stimulation, the state point potentially fails to follow the resting point directly, and moves toward the trajectory of a transient spike to approach the new location of the resting point. This phenomenon is investigated on the FHN-MDS circuit using ramp and step stimuli in Figure 3D.

### 4.2. Adaptive exponential (AdEx) integrate and fire neuron model

The AdEx is a two-dimensional neuron model that mathematically describes the dynamical relationship between the membrane potential of the neuron *v*(*t*) and an adaptation current *w*(*t*). This model is defined by the following system of non-linear ordinary differential equations:

(41) $$\{\begin{array}{l} C\cdot \dot{v}=-g_{L}\cdot (v-E_{L})+g_{L}\cdot \Delta_{T}\cdot \exp(\frac{v-V_{T}}{\Delta_{T}})+I-w\\ \tau_{w}\cdot \dot{w}=a\cdot (v-E_{L})-w \end{array}$$

$$\text{if }v>0\text{ mV then}\{\begin{array}{l} v\leftarrow v_{r}\\ w\leftarrow w_{r}=w+b \end{array}$$

where *C* is total capacitance, *g*_*L*_ is total leak conductance, *E*_*L*_ is effective rest potential, Δ_*T*_ is threshold slope factor, *V*_*T*_ is effective threshold potential, *I* is injected current, *a* is conductance, τ_*w*_ is time constant of the adaptation current, *v*_*r*_ is reset voltage, and *b* is the adaptation reset parameter. One of the important characteristics of the AdEx distinguishing it from the other two dimensional neuron models is that its mathematical parameters have equivalent physiological quantities, because the model has been soundly developed based on a biophysical model of a regular spiking pyramidal cell and its behavior completely fits to neuro-signals recorded from real-life pyramidal cells (Clopath et al., 2007). The first equation of the AdEx is extended based on the LIF neuron models family (Abbott, 1999) that describes the exponential-shaped upraising of the neuron membrane potential during an action potential. Whenever the membrane potential approaches the threshold (*v*_th_), the exponential term in the equation results in a rapid increase of the membrane potential. The downward portion of the spike shape is produced by an auxiliary reset condition. Subthreshold and spike-triggered adaptations are, respectively, considered using the parameters *a* and *b* in the second equation.

According to the explanations in the previous section, the model can be rewritten in the general form of Equation (2) and then mapped easily on the proposed platform. The general form of AdEx neuron model in our mapping is given by:

(42) $$\{\begin{array}{l} x=v,y=w\\ \alpha =1/C,\beta =1/\tau_{w}\\ b=I,c=0\\ F(x)=-g_{L}\cdot (x-E_{L})+g_{L}\cdot \Delta_{T}\cdot \exp(\frac{x-V_{T}}{\Delta_{T}})\\ G(x)=a\cdot (x-E_{L}) \end{array}.$$

Despite the simplicity of this two equation model with only a few parameters, this model can reproduce a wide range of physiological firing patterns. Here, we investigate a number of its main responses.

#### 4.2.1. Tonic spiking

**Figure 4:**
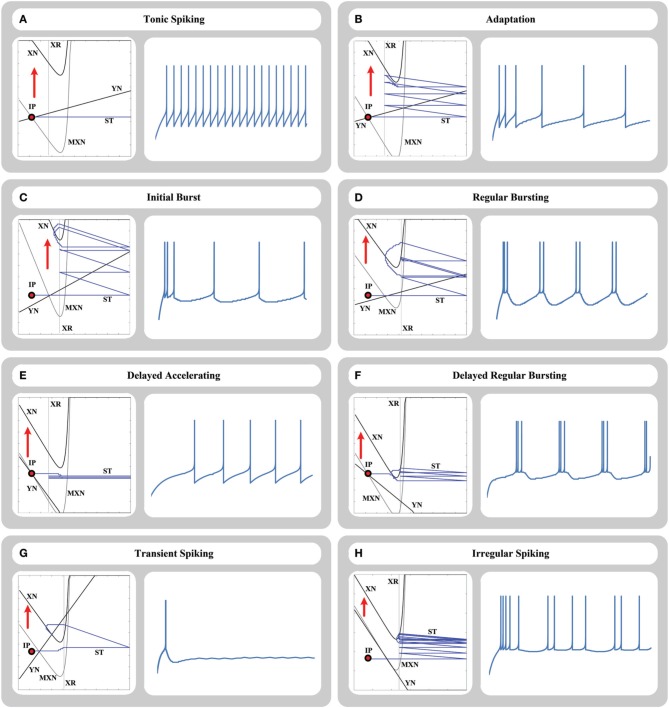
**Phase-plane trajectory and time-domain representation of (A) tonic spiking, (B) adaptation, (C) initial burst, (D) regular bursting, (E) delayed accelerating, (F) delayed regular bursting, (G) transient spiking, and (H) irregular spiking responses for 64-bit memristive AdEx neuron circuit**. In the phase plane, XN and YN are the nullclines, MXN is the momentary location of XN nullcline, ST is the trajectory of the state point in the plane, IP is the initial location of the state point, and XR is the reset line.

In this response, when a step current is injected into the neuron, it starts to fire repetitively, and produces a spike train with a constant frequency in its output. Figure 4A shows the tonic spiking response of our circuit in the phase plane and time domain representations.

#### 4.2.2. Adaptation

In this response, when a step current is injected into the neuron, the neuron starts to fire repetitively and adapt the spike frequency from a relatively high initial frequency to a specific lower frequency. Figure 4B shows the adaptation response of our circuit in the phase plane and time domain representations.

#### 4.2.3. Initial bursting

In this response, when a step current is injected into the neuron, the neuron produces an initial burst of spikes and then starts to fire repetitively with a constant frequency. Figure 4C shows the initial bursting response of our circuit in the phase plane and time domain representations.

#### 4.2.4. Regular bursting

In this response, when a step current is injected into the neuron, the neuron produces a repetitive burst of spikes with a constant frequency (first burst may have different shape due to the initial state point). Figure 4D shows the regular bursting response of our circuit in the phase plane and time domain representations.

#### 4.2.5. Delayed accelerating

In this response, when a step current is injected into the neuron, the neuron produces an initial delay by a slow increase in the membrane potential, and then produces a spike train. The frequency of spike train increases by time as a transient phase (accelerating transient) and then the frequency is fixed. Figure 4E shows the delayed accelerating response of our circuit in the phase plane and time domain representations.

#### 4.2.6. Delayed regular bursting

Similar to the delayed accelerating, in this response, when a step current is injected into the neuron, the neuron produces an initial delay by a slow increase in the membrane potential, and then produces a repetitive burst of spikes with a constant frequency. Figure 4F shows the delayed accelerating response of our circuit in the phase plane and time domain representations.

#### 4.2.7. Transient spiking

In this response, when a step current is injected into the neuron, the neuron produces one transient spike and then remained in the resting state. Figure 4G shows the transient spiking response of our circuit in the phase plane and time domain representations.

#### 4.2.8. Irregular spiking

The AdEx model can represent the irregular spiking behavior, which is a chaotic response, despite this fact that the equations are deterministic. According to this response, inter-spike intervals vary over time without a periodic pattern. Figure 4H shows the irregular spiking response of our circuit in the phase plane and time domain representations.

### 4.3. Izhikevich neuron model

Izhikevich model (Izhikevich, 2003) is one of the well-known neuron models which has been applied to a variety of brain simulation applications. This model is capable of mimicking a wide range of firing patterns and their underlying bifurcation scenarios using a two-dimensional relatively simple phase plane. Its simple equations results in an optimum computational cost to achieve an accurate inclusive group of bio-inspired spike patterns. Two coupled dynamical equations of this model is given by:

(43) $$\{\begin{array}{l} \dot{v}=0.04v^{2}+5v+140-u+I\\ \dot{u}=a\cdot (b\cdot v-u) \end{array}$$

$$\text{if }v>30\text{ mV then}\{\begin{array}{l} v\leftarrow c\\ u\leftarrow u+d \end{array}$$

where the main dynamical variables of the model, *v* and *u*, are, respectively, the membrane potential, and membrane recovery variables of the neuron. Dynamic of the recovery variable *u* models the activation of the ionic channels such as K^+^ and Na^+^ channels, and provides controlling feedback to *v*. Parameters *a, b, c, d* are dimensionless adjusting parameters of the model to achieve various responses and spike patterns. The auxiliary resetting function represented above resets the membrane potential and the recovery variable when the membrane potential reaches its pre-determined apex (*v*_th_ = 30 mV) and neuron fires. This model can be conveniently rewritten in the general form of Equation (2) and then mapped easily on the proposed platform using the procedure explained in the previous sections. The general form of Izhikevich neuron model in our mapping is given by:

(44) $$\{\begin{array}{l} x=v,y=u\\ \alpha =1,\beta =a\\ b=I,c=0\\ F(x)=0.04x^{2}+5x+140\\ G(x)=b\cdot x \end{array}.$$

**Figure 5:**
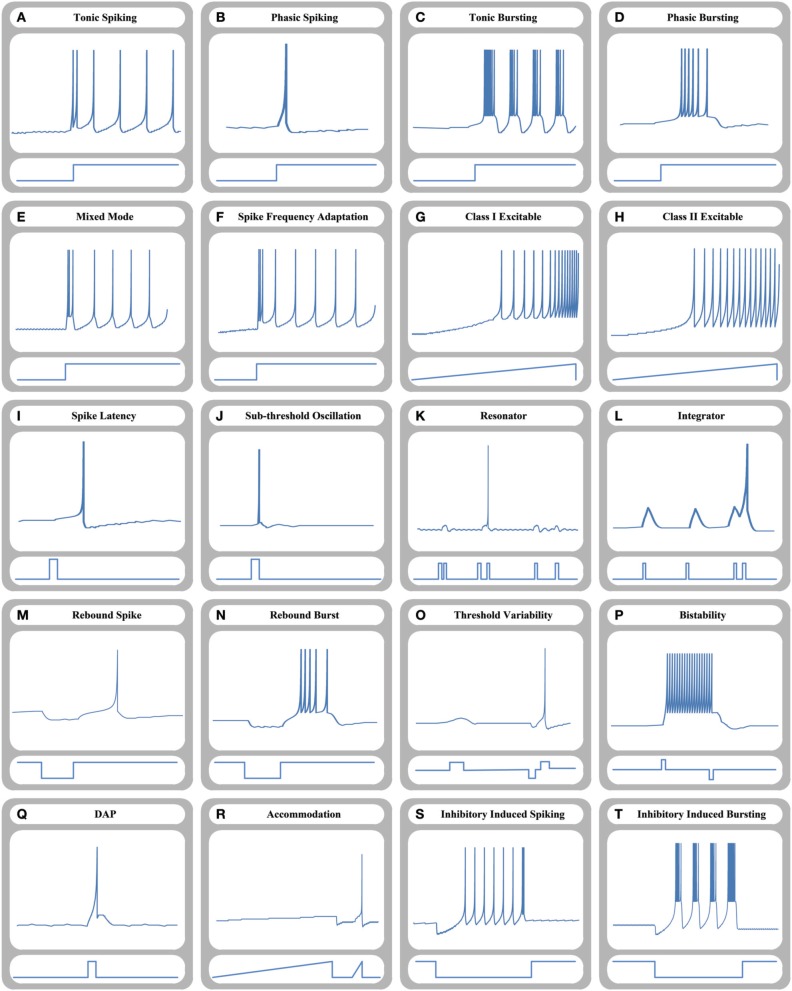
**Time-domain representation of different dynamical behaviors of a 64-bit memristive Izhikevich neuron circuit**. **(A)** Tonic spiking, **(B)** phasic spiking, **(C)** tonic bursting, **(D)** phasic bursting, **(E)** mixed mode, **(F)** spike frequency adaption, **(G)** class I excitable, **(H)** class II excitable, **(I)** spike latency, **(J)** sub-threshold oscillation, **(K)** resonator, **(L)** integrator, **(M)** rebound spike, **(N)** rebound burst, **(O)** threshold variability, **(P)** bistability, **(Q)** Depolarized After-Potential (DAP), **(R)** accommodation, **(S)** inhibitory induced spiking, and **(T)** inhibitory induced bursting.

Time-domain neuron-like responses of the proposed 64-bit memristive cellular Izhikevich-MDS approach are shown in Figure 5. As shown, the circuit can reproduce all key responses of the original Izhikevich neuron model and transitions from resting state to the spiking states by adjusting model parameters and applying special input current shapes.

## 5. Hardware prototype

In this section we introduce a potentiometers-based board simulating our memristive system. This board is remarkably useful for improving the level of understanding about MDS approach, and the concept of learning in this approach for devising compatible learning algorithms, and also developing novel dynamical models with the capability of mimicking new responses. Note that detailed fabrication of this approach on the memristor-crossbar/CMOS platform considering all platform constraints is not the main aim of this paper and may be investigated in the future.

For circuit-level realization of the approach, the essential information about the target dynamical system is mapped into arrays of resistors with different resistances and also a number of logic gates for auxiliary functions such as reset equations. In our general circuit, type of the response and the dynamical system can be changed by changing these two parts of the circuit. Hence, we use the programmable resistors known as multiturn potentiometers and the programmable digital logic gates known as FPGA to develop a fully reconfigurable and generalized hardware.

**Figure 6:**
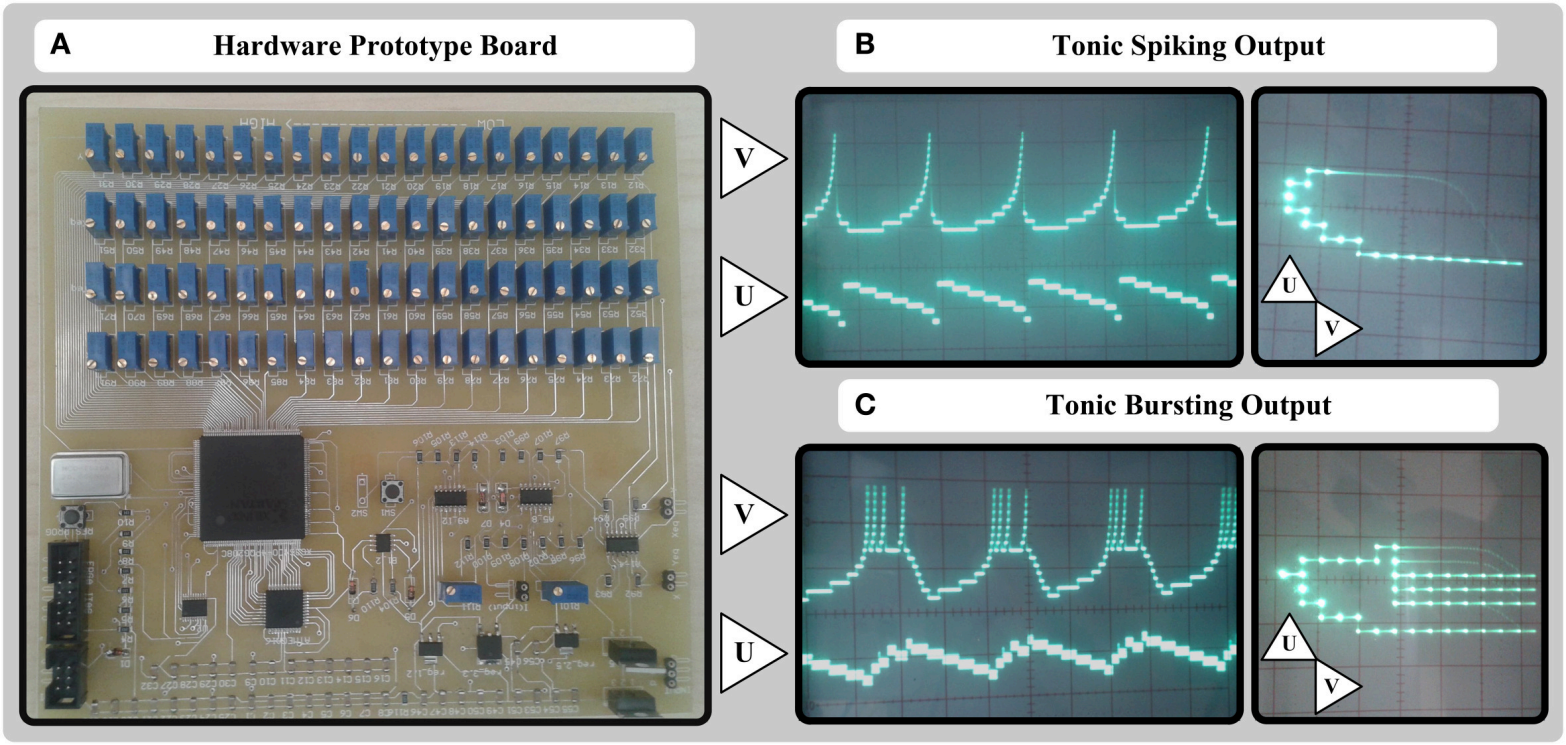
**A simple 20-bit potentiometers-based hardware prototype and its output signals**. **(A)** Hardware board consisting of potentiometer arrays, Opamps, one FPGA, and one AVR microcontroller. **(B)** Output *u* and *v* signals for tonic spiking response of MDS-Izhikevich model. **(C)** Output *u* and *v* signals for tonic bursting response of MDS-Izhikevich model

The final board for a 20 × 20 cellular approach is depicted in Figure 6A, and *v* and *u* analog output signals of the circuit for tonic spiking and tonic bursting responses of Izhikevich neuron model are depicted in Figures 6B,C, respectively. As shown in the figure, we used 4 × 20 = 80 100 kΩ multiturn potentiometers instead of memristors and TL074 opamps with *R*_*f*_ = 10 kΩ to implement X_DAC, Y_DAC, X_eqa, Y_eqa units of the circuit shown in Figure 2. According to Equations (21) and (25), we ought to evaluate the value of *G*_mx0_, *G*_my0_, *A*_*x*_, *A*_*y*_ based on our board voltage level and other constraints such as resistance values before we go through the detailed circuit design. In this case, knowing *R*_*f*_ = 10 kΩ, *v*_*d*_ = 3.3 V, and the resistance range of the potentiometers, we decided to limit the range of variable resistances to (10–80 kΩ). Therefore, we can easily evaluate the above-mentioned parameters in Equations (21) and (25) by checking the lower boundary (*i* = 0) and upper boundary (*i* = 19) conditions:

(45) $$\{\begin{array}{l} G_{\text{mx}}(0)=(A_{x}.(0)+1)\cdot G_{\text{mx0}}=\frac{1}{80\times 10^{3}}\\ G_{\text{mx}}(19)=(A_{x}.(19)+1)\cdot G_{\text{mx0}}=\frac{1}{10\times 10^{3}} \end{array}$$

(46) $$\Rightarrow \{\begin{array}{l} G_{\text{mx0}}=G_{\text{my0}}=\frac{1}{80\times 10^{3}}\\ A_{x}=A_{y}=\frac{7}{19} \end{array}.$$

The gain of other adders, subtractors, and op-amp based absolute value circuits are equal to unit. Also the sign of the velocities are calculated using single-supply TLC272 opamps working in the saturation mode, and comparing the velocity voltage values with the ground voltage. The final absolute values are converted to 8-bit digital values using two built-in ADC of ATmega16 microcontroller, and sent to the Spartan3 XC3S400-4PQ208C FPGA. The VCO units, the state registers, and the auxiliary reset equations are synthesized on the FPGA, and 2 × 20 wires of state registers are connected back from the FPGA to the input of potentiometer arrays.

## 6. Error analysis

One of the challenging topics in dynamical systems is error analysis of an approximation of the system such as piece-wise linear approximations (Soleimani et al., 2012) and cellular mappings (Bavandpour et al., 2014) in comparison with the original system. This challenge is raised because of two main reasons: first, there are different expectations from different dynamical systems which change the fidelity factors in error analysis, and the concept of error ought to be redefined by these expectations. For example, in a neuron model, these different fidelity factors include exact timing of the spikes, rate of the spikes, exact trajectory of the spikes and refractory phases, etc. Second, the equations and parameters of dynamical systems are sensitive, and a small change in them may cause a fundamental change in the whole behavior (bifurcations) of the system. In this section, we assess error in terms of both trajectory in the phase plane and the timing of output spikes for our approach.

In the Section 2, in the first step, we changed the representation of the general form of the target dynamical system, and showed that the velocity of a given state point in the phase plane is related to the vertical distance of that point from the nullclines. This step was just a change in the representation form that did not cause any kind of error in the system. Therefore, the cellular mapping, and different variations in fabrication process are the source of error in our system. The detailed circuit implementation of different blocks of the system and investigation of the hardware variations in the system is not in the scope of this paper, and will be studied as a future work. So, here we investigate the effect of cellular mapping.

**Figure 7:**
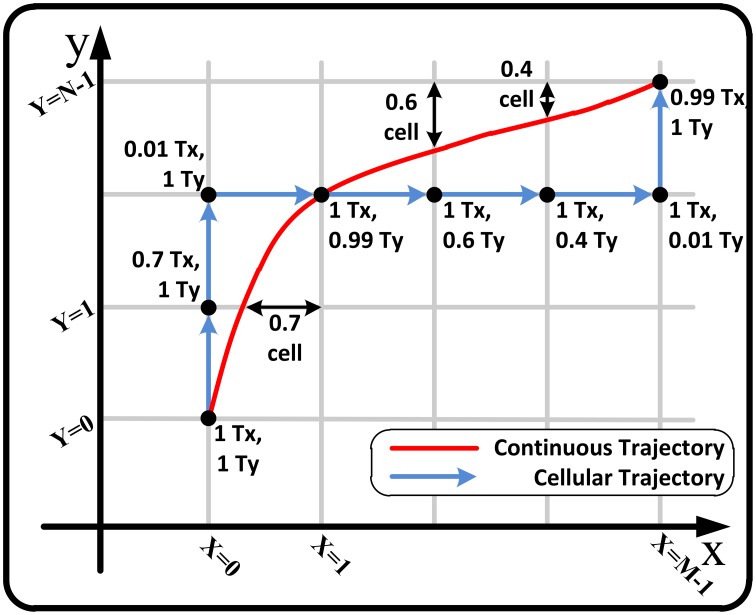
**A sample continuous trajectory in the phase plane and its proportional cellular trajectory using the motion times**.

As explained before, although our approach turns the continuous phase plane into a plane with discrete crossbar cells, it can exactly locate any point in the plane and indicate that point using the motion times. It implies that our asynchronous approach can track any trajectory in the plane with the sufficient number of cells. Figure 7 shows a sample continuous trajectory in the phase plane and its proportional cellular trajectory using the motion times. In the figure, the motion times (*T*_*x*_, *T*_*y*_) for each cell are represented. These times are updated in each cell based on Equations (9) and (11). As shown, the virtual cellular trajectory (cellular trajectory + motion times) can exactly track the continuous trajectory in the phase plane under specific conditions on the trajectory and the number of cells.

Note that the Figure 7 represents the ideal condition where the velocity in the intra-cells space is approximately constant and consequently the trace is smooth. In our approach, we calculate the velocity for the cross point of the cells and consider it to be constant over intra-cell space. This matter causes error in form of momentary and permanent lag, lead and deviation in tracking where the velocity changes are more erratic and uneven. This effect can be significantly reduced by increasing the number of cells. Note that the injected currents bb, cc given in Equation (28) are directly influence the velocity and do not cause this kind of error.

**Figure 8:**
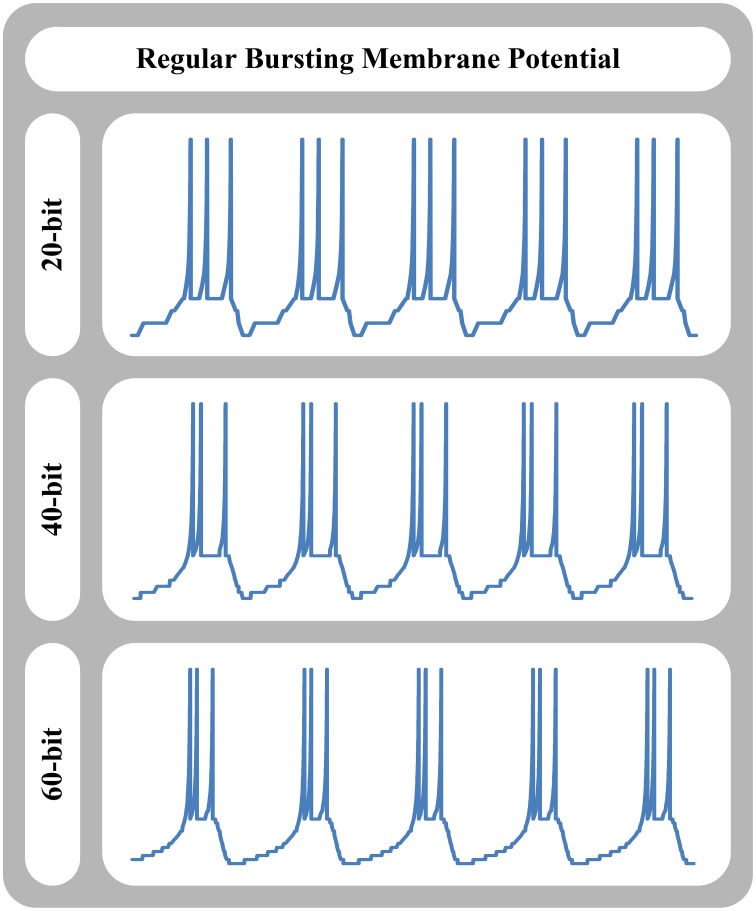
**Signal shape of regular bursting produced by MDS-AdEx approach using 20, 40, and 60 bits**.

Here, we separate spike timing error caused by the piece-wise constant velocity nature, and spike shape error caused by cellular nature of our approach. Table 2 shows the spike timing error and spike energy error of our approach in comparison with the original model for tonic spiking and regular bursting responses of different neuron models such as the FitzHugh-Nagumo, AdEx, and Izhikevich neuron models. For the spike timing error, we calculated the relative error of duration of spike train (duration of one limit cycle). Also, for the spike energy error, we synchronized the spike trains and calculated the relative error for the energy of signal shapes for one cycle. As can be seen, absolute value of the spike timing error is decreased by increasing the number of bits or cells in the system. Note that the relative timing of the signals (lead/lag) is changed randomly. Moreover, the relative spike energy error is decreased by increasing the number of bits or cells in the system. Figure 8 shows the signal shape of regular bursting produced by our MDS-AdEx using 20, 40, and 60 bits.

**Table 2:** **The relative spike timing error and relative spike energy error of our approach in comparison with the original model for tonic spiking and regular bursting responses of the FitzHugh-Nagumo, AdEx, and Izhikevich neuron models**.

**Neuron model**	**Response type**	**20-bit (%)**	**40-bit (%)**	**60-bit (%)**	**80-bit (%)**	**100-bit (%)**
**(A) RELATIVE SPIKE TIMING ERROR**						
FHN	Tonic spiking	1.78	1.04	0.67	0.43	0.26
AdEx	Tonic spiking	2.29	1.34	1.00	0.79	0.54
	Regular bursting	3.52	1.73	1.08	0.81	0.65
Izhikevich	Tonic spiking	2.03	1.22	0.88	0.54	0.32
	Regular bursting	3.01	1.69	1.01	0.76	0.55
**(B) RELATIVE SPIKE ENERGY ERROR**						
FHN	Tonic spiking	3.24	1.78	1.22	0.88	0.62
AdEx	Tonic spiking	9.41	5.09	3.99	2.98	2.07
	Regular bursting	17.55	8.77	5.04	4.57	3.95
Izhikevich	Tonic spiking	7.85	4.08	3.12	2.01	1.44
	Regular bursting	10.14	5.00	3.85	2.97	2.45

## 7. Remarks

This paper presented a novel, efficient, fully reconfigurable approach for implementing neuromorphic dynamical systems. This approach shows a huge capability in different aspects, and it can be developed and fabricated for different applications. In this section, we present two main capabilities of our approach and attempt to point out an acceptable roadmap for research works toward which we can obtain a powerful hardware for neuromorphic applications. These two capabilities, which are the key topics in neuromorphic engineering, are (1) configuring and networking, and (2) learning.

### 7.1. Configuring and networking capability

Considering various applications of the neural systems, it can be easily concluded that any proposed approach for hardware implementation of a single neural element ought to provide the capability of networking with variable weights. Here, we draw a roadmap to achieve a reconfigurable network of Memristive Dynamical System (MDS) cells with inherent plasticity. As explained, MDS approach produces both analog and digital one-hot outputs, and accepts analog voltage as injected input to the system. This feature makes it feasible to replace conventional hardware implementation of single neural cells with the MDS cells in a wide range of applied digital/analog weighted networking schemes with plasticity to achieve a network of optimum accurate general neural cells. Besides, it is convenient to propose novel networking schemes to connect Memristive Dynamical System (MDS) cells with variable weights, so the resultant system is flexible network of general neural cells with variable intera-cell dynamics and the capability of applying bio-inspired learning schemes.

**Figure 9:**
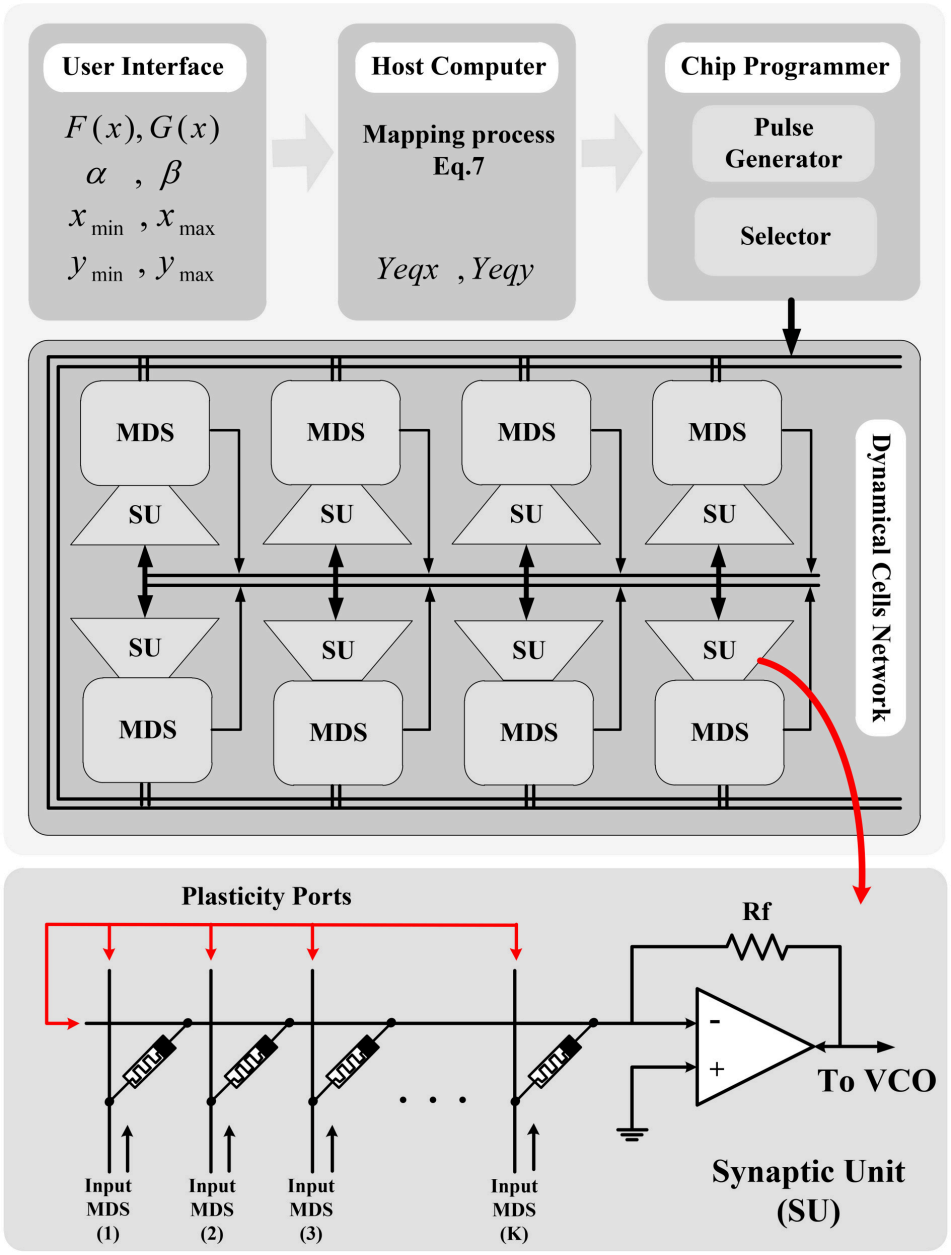
**Conceptual network-level structure for networking the MDS cells**. The user inputs required mathematical information of the model into the host computer, and the required equilibrium curves data are calculated and programmed into the neuro-chip memristors using a programmer unit.

Figure 9 shows the overall conceptual structure of the system which contains the network of the proposed Memristive Dynamical System (MDS) cells connecting to each other through a weighting and integrating Synaptic Unit (SU) and sharing a connection bus for programming the reconfigurable MDS cells. In this conceptual model of the system, the user is provided with a user interface to determine the equilibrium functions and the desired intervals [*x*_min_, *x*_max_) and [*y*_min_, *y*_max_) on the dynamical variables *x* and *y*. A host processor calculates the equilibrium arrays *Y*_eqx_ and *Y*_eqy_ and their equivalent memristances (where *b* = *c* = 0), and applies a compatible programmer system to program them on the stand-alone neuro-chip using a developed communication protocol. During this process, the equilibrium memristor arrays are selected and programmed using the special pulse shapes proposed for writing into the memristors. Clearly, the reconfigurable memristors ought to be updated every time the dynamical system parameters are changed. Then, the MDS cells starts to track their deterministic trajectory in the phase plane according to the moving policy given by Equations (14–17) implemented on the neuro-chip.

### 7.2. Learning

**Figure 10:**
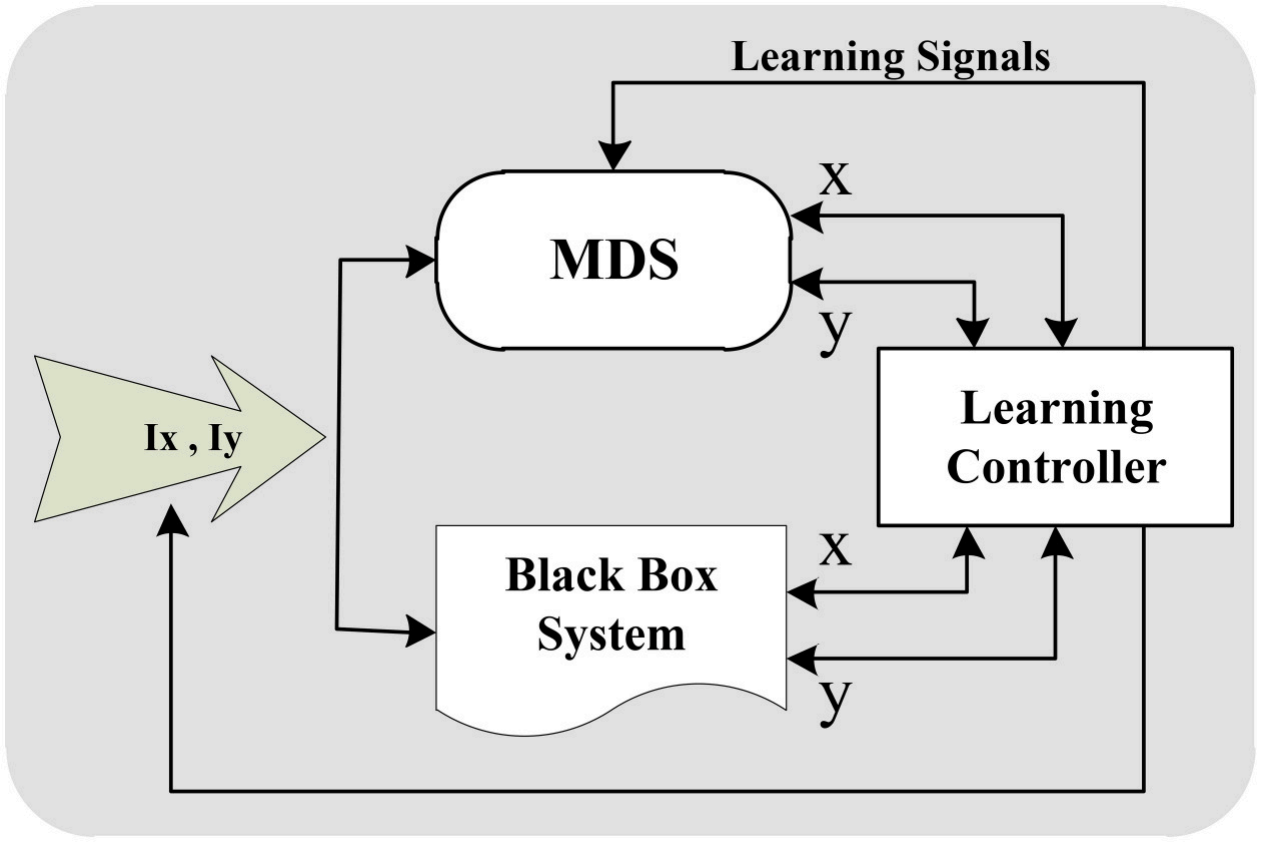
**A possible learning system block diagram which extracts the behavior of an unknown black box system and teaches an MDS cell to imitate this behavior**.

Applying the flexible memristors in a well-mapped general hardware brings a significant advantage of learning capability to the MDS approach. In other words, the MDS is capable of learning various intra-cell dynamics with various complexities to reproduce various signal shapes specifically spike patters in its output. One of the major advantages of our novel MDS approach over previous CMDS (Bavandpour et al., 2014) is our novel mapping which uses the vertical distance of the state point from the nullclines to calculate the velocity. In this mapping, the nullclines while in the CMDS the memristors store the value of velocity in all over the cellular phase plane. Therefore, learning process in MDS is significantly simpler and more effective than CMDS. Considering this feature, different intra-cell learning schemes can be applied to the MDS to teach it different dynamical behaviors with no straight knowledge on the dynamical equations. Figure 10 shows the conceptual block diagram of a possible learning system for MDS. As shown, target system is a black box system with unknown mathematical model governing its dynamical behavior. Assume that the system can be modeled with a two-dimensional dynamical system, and the state variables of the black box are observable. In this condition, the dynamical behavior of the system can be extracted by applying different stimulus inputs in different initial states. Output signals of the black box can be applied to MDS system as teacher signals to follow its behavior. Learning controller detects the black box state, and modifies MDS behavior in the same state using teacher signal. Modification of MDS behavior during the learning phase is done by changing the memristance of the memristors using learning signals.

## 8. Discussion

This study presented an unconventional computing approach based on a novel general mapping for dynamical systems in two-dimensional cellular phase space, and then its hardware implementation on efficient hybrid memristors-crossbar/CMOS memristive circuit. The proposed approach calculates the velocity vector using the vertical distance of the state point from the nullclines, and applies an unconventional VCO-based asynchronous technique to track the state trajectory in the phase space. This approach employed 4*n* memristors and no switch for implementing an *n*-cell system in comparison with the 2·*n*^2^ memristors and 2*n* switches of a Cellular Memristive Dynamical System (CMDS). In addition, our proposed circuit provides both analog and one-hot digital values of dynamical variables offering a wide range of choices for interconnections and networking schemes. The fidelity of MDS approach was investigated on widely used two-dimensional neuromorphic dynamical systems such as the FitzHugh-Nagumo model, the Adaptive Exponential (AdEx) integrate and fire model, and the Izhikevich neuron model covering a wide range of real-life neuronal dynamic behaviors and responses. The MDS-based neurons were simulated with various stimulus currents, and it was showed that they can mimic every key response produced by the original models. Moreover, we clarified different definitions of error in neuromorphic dynamical systems and investigated spike timing error and spike shape error for different responses of different neuron models. The error results showed that our approach can properly mimic the exact behavior of the dynamical system. In conclusion, the key advantages of the proposed approach can be listed as:

- It is general and highly programmable.
- It achieves a relatively high accuracy in a low resolution cellular space.
- Its implementation cost is almost independent from the computational effort of the target mathematical model.
- It is capable of implementing mathematically indescribable dynamical systems.
- It is implementable on a nanoscale efficient memristor/CMOS hardware platform.
- It is conveniently feasible to apply conventional analog or digital networking schemes to the MDS cells, and also propose novel networking schemes.
- It is capable of learning unknown intra-cell dynamics.

Toward the future roadmap, the problems which ought to be solved can be listed as:

- A detailed circuit design and analysis process to achieve an optimum circuit for different blocks of the MDS system.
- Developing unconventional learning methods for MDS-based dynamical systems.
- A novel compatible mathematical method for stability analysis of the proposed cellular mapping.

## Funding

This work was partially funded by EU HBP project under grant number FP7-ICT-2013-FET-F-604102, by EU H2020 ECOMODE project under grant agreement 604102, and by Spanish research grant (with support from the European Regional Development Fund) TEC2012-37868-C04-01 (BIOSENSE).

### Conflict of interest statement

The authors declare that the research was conducted in the absence of any commercial or financial relationships that could be construed as a potential conflict of interest.
